# Chimeric cells of maternal origin do not appear to be pathogenic in the juvenile idiopathic inflammatory myopathies or muscular dystrophy

**DOI:** 10.1186/s13075-015-0732-0

**Published:** 2015-09-04

**Authors:** Carol M. Artlett, Sihem Sassi-Gaha, Ronald C. Ramos, Frederick W. Miller, Lisa G. Rider

**Affiliations:** Department of Microbiology and Immunology, Drexel University College of Medicine, 2900 Queen Lane, Philadelphia, PA 19129 USA; Department of Medicine, Thomas Jefferson University, 1020 Walnut Street, Philadelphia, PA 19107 USA; National Institute of Environmental Health Sciences, National Institutes of Health, Department of Health and Human Services, Environmental Autoimmunity Group, Program of Clinical Research, 9000 Rockville Pike, Bethesda, MD 20892 USA; Present address: Agis Global, Business Development Executive, 1266 East Main Street, Stamford, CT 06902 USA

## Abstract

**Introduction:**

Microchimeric cells have been studied for over a decade, with conflicting reports on their presence and role in autoimmune and other inflammatory diseases. To determine whether microchimeric cells were pathogenic or mediating tissue repair in inflammatory myopathies, we phenotyped and quantified microchimeric cells in juvenile idiopathic inflammatory myopathies (JIIM), muscular dystrophy (MD), and noninflammatory control muscle tissues.

**Method:**

Fluorescence immunophenotyping for infiltrating cells with sequential fluorescence in situ hybridization was performed on muscle biopsies from ten patients with JIIM, nine with MD and ten controls.

**Results:**

Microchimeric cells were significantly increased in MD muscle (0.079 ± 0.024 microchimeric cells/mm^2^ tissue) compared to controls (0.019 ± 0.007 cells/mm^2^ tissue, *p* = 0.01), but not elevated in JIIM muscle (0.043 ± 0.015 cells/mm^2^). Significantly more CD4+ and CD8+ microchimeric cells were in the muscle of patients with MD compared with controls (mean 0.053 ± 0.020/mm^2^ versus 0 ± 0/mm^2^*p* = 0.003 and 0.043 ± 0.023/mm^2^ versus 0 ± 0/mm^2^*p* = 0.025, respectively). No differences in microchimeric cells between JIIM, MD, and noninflammatory controls were found for CD3+, Class II+, CD25+, CD45RA+, and CD123+ phenotypes, and no microchimeric cells were detected in CD20, CD83, or CD45RO populations. The locations of microchimeric cells were similar in all three conditions, with MD muscle having more microchimeric cells in perimysial regions than controls, and JIIM having fewer microchimeric muscle nuclei than MD. Microchimeric inflammatory cells were found, in most cases, at significantly lower proportions than autologous cells of the same phenotype.

**Conclusions:**

Microchimeric cells are not specific to autoimmune disease, and may not be important in muscle inflammation or tissue repair in JIIM.

## Introduction

The role of microchimeric cells in health and disease has been controversial. Microchimeric cells are acquired during pregnancy, with the transfer of cells from fetus to mother or mother to fetus. Microchimeric cells have been documented to be elevated in the peripheral blood and affected tissues of patients with autoimmune diseases, such as systemic sclerosis [1], systemic lupus erythematosus [2], neonatal lupus [3], and juvenile idiopathic inflammatory myopathies (JIIM) [4–6]. Recently, microchimeric cells were found to be elevated in specific target tissues, such as the liver in hepatitis C infection [7] and tumors, such as HER2-positive breast cancer, cervical, lung and thyroid cancer, and melanoma [8–10]. However, not all studies have documented higher levels of chimeric cells in autoimmune conditions [11] or cancer [12]. This variability suggests that they might be recruited nonspecifically to sites of inflammation and tissue injury [13, 14] or participate in tissue repair [8].

The JIIM are systemic autoimmune diseases characterized by chronic muscle inflammation and weakness. Juvenile dermatomyositis (JDM), the form of JIIM with characteristic photosensitive skin rashes, including Gottron’s papules and heliotrope rash, is the most common of the JIIM and is thought to be mediated by CD4+ T cells, B cell and dendritic cell attack on muscle capillaries, whereas juvenile polymyositis (JPM), the form of JIIM without characteristic rashes, is thought to be mediated by CD8+ T cells on myofibers [15–17]. We previously found elevated levels of maternal microchimeric cells in muscle biopsies and peripheral blood of boys with JIIM and characterized these cells to be in the CD4+ and CD8+ peripheral T cells [4]. However, the phenotypes of the microchimeric cells in affected muscle tissues were not investigated. The current study analyzes the frequency of microchimeric cells within different inflammatory phenotypes in the muscle of JIIM and compares these findings with the nonautoimmune inflammatory muscle disorder muscular dystrophy (MD) [18] and with noninflammatory control (NIC) muscle tissue. We sought to determine whether microchimeric cells have a pathogenic or reparative role in JIIM.

## Methods

### Patients

All studies were performed with full Institutional Review Board approval from the National Institutes of Health and waived approval from the Institutional Review Board at Drexel University College of Medicine. All patients consented to the study. Muscle biopsies were obtained for diagnosis, and prior to initiation of therapy, from ten patients with JIIM (six JDM, four JPM), nine MD (eight Duchenne, one Becker dystrophy) and ten controls without inflammatory disease (four mitochondrial myopathies, six histologically normal) and analyzed by immunofluorescence for specific phenotypes and by fluorescence in situ hybridization (FISH) for maternal microchimeric cells. The ages of the JIIM patients ranged from 3 to 16 years [15, 16], the patients with MD from 2 to 14 years [19], and the controls from 2 to 17 years. All tissues were paraffin embedded and derived from males and were cut at 5 μM. No patient had prior blood transfusions. The size of the tissue sample ranged from 9 to 96 mm^2^ in JIIM, 6 to 245 mm^2^ in MD, and 20 to 62 mm^2^ in the controls.

### Immunofluorescence/fluorescence in situ hybridization

Before immunofluorescent staining was performed, one slide from each biopsy was stained with hematoxylin and eosin to verify the histological appearance of the tissue and the presence of inflammatory cells. Biopsies were selected based on infiltration density. The immunofluorescent assessment of the tissues was performed first, and all positive cells of each immunophenotype were documented. Tissues were stained for T cells (CD3, CD4, and CD8), T cell activation markers (CD25 and HLA Class II), memory T cells (CD45RO), naïve T cells (CD45RA), B cells (CD20) and dendritic cells (CD123 and CD83) by using antibodies from Neomarkers (Fremont, CA, USA) or Dako (Carpinteria, CA, USA). The secondary antibody carrying the Cy-2 conjugate (Jackson ImmunoResearch, West Grove, PA, USA) was used to detect the primary antibody, and sections were mounted with DAPI and viewed with a Nikon epi-fluorescent microscope with triple-band filter at 1000× magnification (Nikon Instruments, Melville, NY, USA). Positive cells were documented, and sections were processed for FISH analysis as we described previously [1]. Cells that had XX or XY nuclear probes were quantified. The sections were also assessed after FISH for other nuclei that were carrying XX probes but were negative for immunofluorescent stain. Muscle fiber nuclei were documented by morphological analyses of the muscle fiber based on the location and shape of the nucleus within the fiber. The myogenic origin was confirmed in 12 sections by actin staining. Personnel involved with muscle biopsy studies were blinded to patient diagnosis.

Characterization of microchimeric or autologous cells was determined only when both probes were evident in the nucleus (XX for microchimeric cells and XY for autologous cells). We performed immunofluorescence prior to FISH and stained the protein green. Because some immunofluorescence signal can remain after the FISH procedure, we selected red for the X chromosome. This approach left no doubt about the presence of a microchimeric cell, even if residual immunofluorescence staining was present. Biopsy sections were selected to limit the number of overlapping nuclei in the inflammatory cell infiltrates, because overlapping nuclei could result in false assignment of microchimerism due to two X chromosome probes appearing to be in the same nucleus when they are in separate nuclei. Prior to making the final assessment as to whether a cell was microchimeric or not, the entire nucleus of the cell was assessed for the presence of other probes, i.e., possible nuclei lying underneath. Only when it was confirmed that no additional probes were present, were the nuclei assigned as being microchimeric or autologous. Twelve (JIIM or MD) biopsies with many overlapping infiltrating cells without well-defined nuclei were excluded. With this careful approach to immunophenotyping and FISH we reduced the chance of over-identifying microchimeric cells due to overlapping cells and to fluorescent protein remaining after the phenotyping.

### Statistical analyses

Results obtained by FISH were expressed as mean ± standard error of the mean. Differences in the frequency of positive microchimeric cells in the tissue were evaluated using Prism 5 software (GraphPad Software, San Diego, CA, USA). For nonparametric data, the Mann–Whitney rank sum *t* test was used to compare quantities of microchimeric cells. A *p* value ≤ 0.05 was considered significant; due to small sample sizes, *p* = 0.051–0.06 was considered to demonstrate a trend toward significance.

## Results

### Are microchimeric cells enriched in inflammatory diseases and what are their phenotypes?

**Fig. 1:**
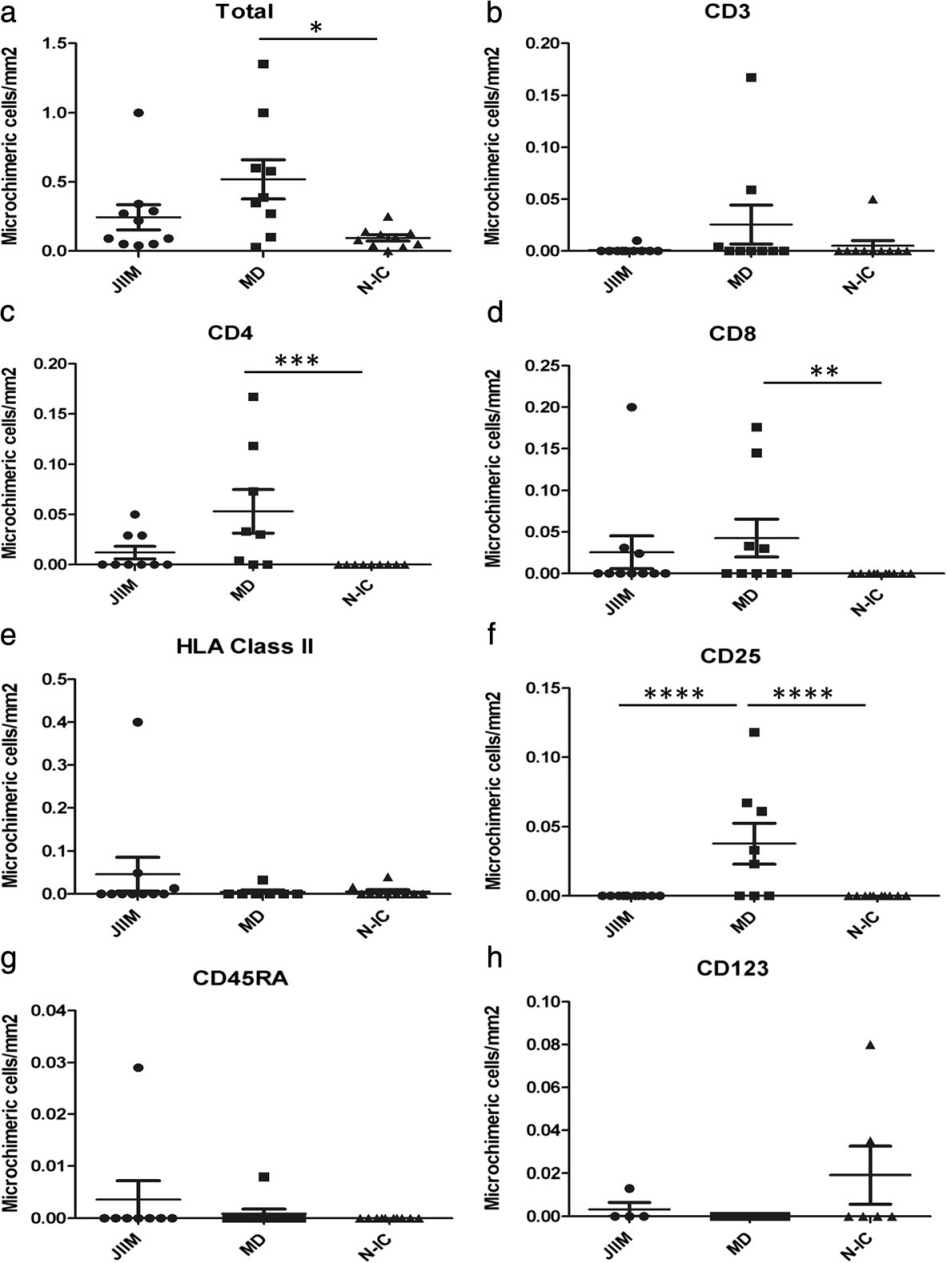
Density of microchimeric cells by immunophenotype in the muscle of patients with juvenile idiopathic inflammatory myopathies (JIIM), muscular dystrophy (MD) and noninflammatory controls (N-IC). Data are presented as mean ± standard error of the mean for each immunophenotype. No microchimeric cells were identified that were positive for CD20, CD45RO or CD83 (data not shown). *JIIM* juvenile idiopathic inflammatory myopathy (*n* = 10), *MD* muscular dystrophy (*n* = 9), *N-IC* noninflammatory controls (*n* = 10). ^*^ *p* = 0.01; ^**^ *p* = 0.025; ^***^ *p* = 0.003; ^****^ *p* = 0.006. (**a**) Total microchimeric cells in tissues; (**b**) CD3 positive microchimeric cells; (**c**) CD4 positive microchimeric cells; (**d**) CD8 positive microchimeric cells; (**e**) HLA class II positive microchimeric cells; (**f**) CD25 positive microchimeric cells; (**g**) CD45RA positive microchimeric cells; (**h**) CD123 positive microchimeric cells

Microchimeric cells were detected in nine of ten JIIM muscle biopsies, nine of nine MD biopsies, and eight of ten NIC biopsies (ten sections per biopsy). Of the total phenotyped and nonphenotyped microchimeric cells across all sections examined, the density was 0.043 ± 0.015 microchimeric cells/mm^2^ of muscle tissue in JIIM, 0.079 ± 0.024/mm^2^ of tissue in MD, and 0.019 ± 0.007/mm^2^ of tissue in NICs (Fig. 1a). MD biopsies had significantly more microchimeric cells/mm^2^ than controls (*p* = 0.01). There was a trend toward more microchimeric cells/mm^2^ of muscle tissue in MD patients compared to JIIM (*p* = 0.06), but there was no difference in the density of microchimeric cells between JIIM and controls.

**Fig. 2:**
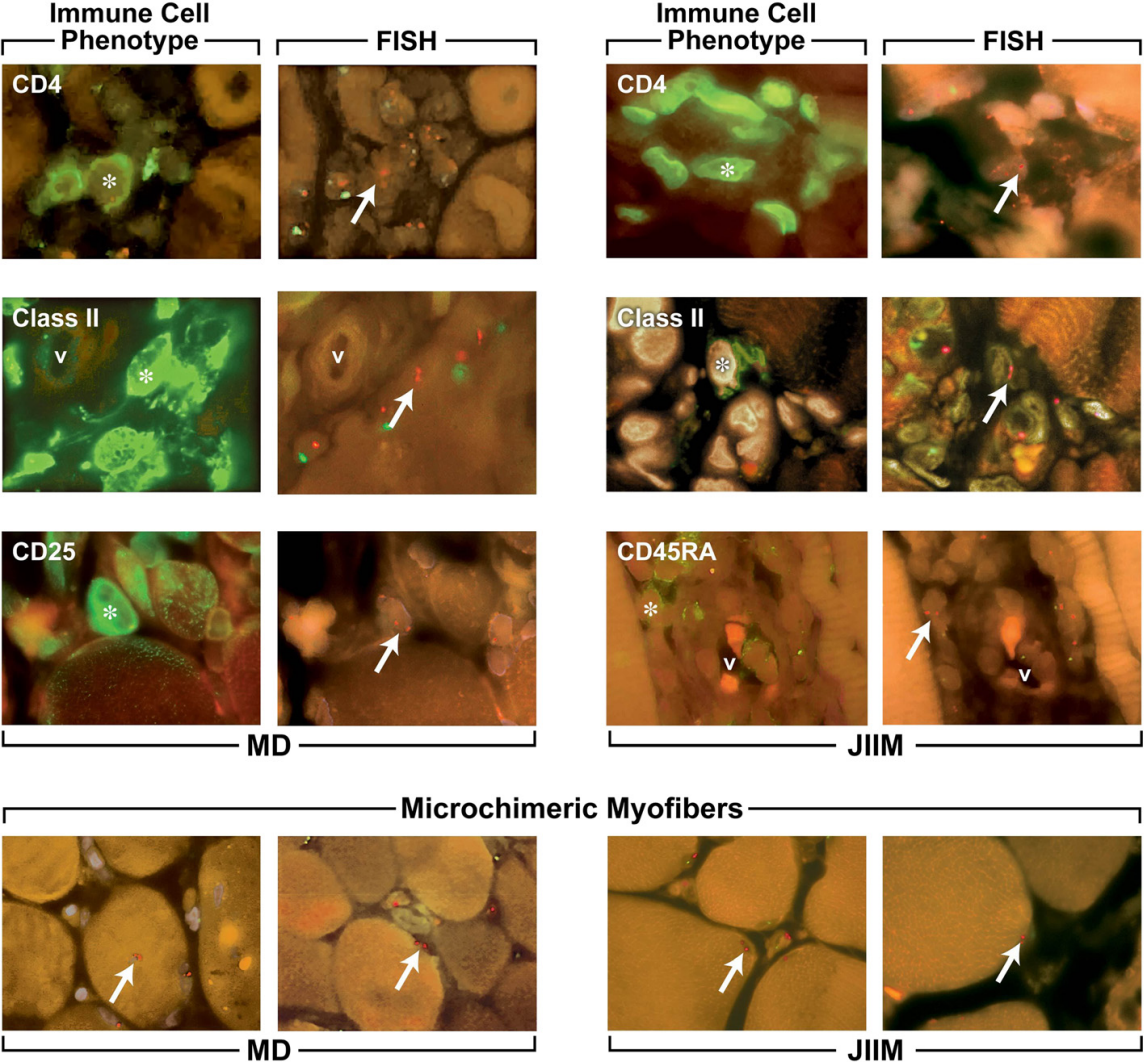
Representative microchimeric cells that are expressing an immunophenotype in the muscle of patients with muscular dystrophy and juvenile idiopathic inflammatory myopathies. Examples of microchimeric cells that express a phenotype are depicted, the *left panel* in each pair is the phenotype and the *right panel* is the corresponding FISH result for that cell. *JIIM* juvenile idiopathic inflammatory myopathy, *MD* muscular dystrophy, *V* vessel. *Star* denotes the phenotype-positive cell (*green*) and the *arrows* indicate the XX probes (*red*) within that phenotyped cell

There were few differences in the phenotypes of the microchimeric cells and no differences in the concentration of microchimeric cells/mm^2^ of muscle tissue in CD3+, Class II+, CD45RA+ or CD123+ phenotypes in patients with JIIM, MD or the controls (Fig. 1b, e, g, h). There were significantly more CD4+ and CD8+ microchimeric cells in the muscle of patients with MD compared with controls (0.053 ± 0.020/mm^2^ vs. 0 ± 0/mm^2^ for CD4+ microchimeric cells [Fig. 1c, *p* = 0.003] and 0.043 ± 0.023/mm^2^ vs. 0 ± 0/mm^2^ for CD8+ microchimeric cells [Fig. 1d, *p* = 0.025]). There was a trend toward more CD4+ microchimeric cells in MD biopsies compared with JIIM (0.053 ± 0.020/mm^2^ vs. 0.012 ± 0.006/mm^2^, *p* = 0.06); whereas the number of CD4+ microchimeric cells did not differ between JIIM and controls. The number of CD8+ microchimeric cells did not differ in the muscle tissue of patients with MD and JIIM, or JIIM and controls. MD muscle tissue had significantly more microchimeric CD25+ cells/mm^2^ of tissue (0.038 ± 0.014/mm^2^), whereas JIIM patients and controls did not have any detectable CD25+ microchimeric cells (Fig. 1f). None of the biopsies had microchimeric cells that were CD20+, CD45RO+ or CD83+ (not shown). Examples of phenotyped microchimeric cells are depicted in Fig. 2.

There were few differences in concentration of microchimeric cells/mm^2^ in the JIIM subsets (not shown). JPM had more CD8+ microchimeric cells/mm^2^ than JDM (0.064 + 0.046/mm^2^ vs. 0 ± 0/mm^2^; *p* = 0.025) and controls (0.064 + 0.046/mm^2^ vs. 0 ± 0/mm^2^, *p* = 0.004) but had similar numbers of CD8+ microchimeric cells/mm^2^ as MD (*p* = 0.51). CD4+ microchimeric cells were detected in JDM, but not in JPM, muscle (0.022 ± 0.009/mm^2^ vs. 0 ± 0/mm^2^), but the concentrations did not differ significantly (*p* = 0.31).

### Does the location of microchimeric cells differ among inflammatory muscle diseases?

**Fig. 3:**
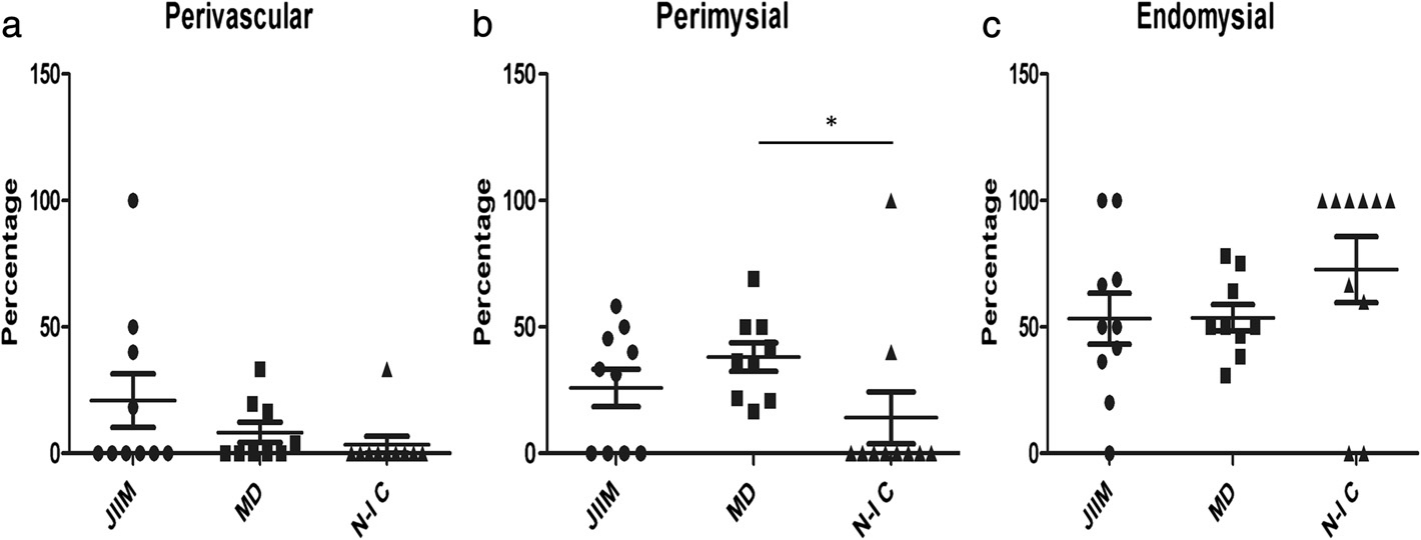
Percentage of microchimeric cells at various muscle locations. Data are presented as mean of the percentage ± standard error of the mean at each location for each diagnostic group. *JIIM* juvenile idiopathic inflammatory myopathy (*n* = 10), *MD* muscular dystrophy (*n* = 9), *N-IC* noninflammatory controls (*n* = 10). ^*^ *p* = 0.014. (**a**) Perivascular location; (**b**) Perimysial location; (**c**) Endomysial location

Overall, 62.2 ± 5.7 % of all microchimeric cells were in the endomysial region, 26.5 ± 4.9 % in the perimysial regions and 11.3 ± 4.2 % in the perivascular regions. There were no significant differences between the percentages of microchimeric cells in the perivascular regions of JIIM or MD patients or the NIC subjects (20.8 ± 10.6 % in JIIM, 8.2 ± 4.1 % in MD, and 3.7 ± 3.5 % in controls; Fig. 3a). In the perimysial regions, microchimeric cells occurred in similar proportions between JIIM and MD patients (25.8 ± 7.4 % vs. 38.1 ± 5.6 %, *p* = 0.62) and between JIIM and controls (25.8 ± 7.4 % vs. 15.6 ± 10.8 %, *p* = 0.14). However, biopsies from MD patients had a higher percentage of microchimeric cells in the perimysial region than controls (33.9 ± 5.5 % vs. 14.0 ± 10.3 %, *p* = 0.014; Fig. 3b). There were no differences in the endomysial regions between JIIM or MD patients or controls (53.3 ± 10.1 % in JIIM, 53.7 ± 5.3 % in MD, and 80.7 ± 10.8 % in controls; Fig. 3c).

The subsets of JIIM (not shown) did not differ in the percentages of microchimeric cells in the perivascular regions (14.6 ± 11.0 % in JPM vs. 25.0 ± 17.1 % in JDM, *p* = 0.90), perimysial regions (41.7 ± 4.6 % in JPM vs. 15.3 ± 10.2 % in JDM, *p* = 0.15) and endomysial regions (43.8 ± 11.9 % in JPM vs. 59.7 ± 15.6 % in JDM, *p* = 0.51).

### Do microchimeric cells drive disease?

For each phenotype we determined whether microchimeric cells were enriched as a percentage of all microchimeric cells, compared to autologous cells of that phenotype as a percentage of all autologous immunophenotyped cells. Muscle cells were excluded in this analysis. An enrichment of microchimeric cells compared to autologous cells could indicate their involvement in disease pathogenesis. In most cases, the proportion of microchimeric cells of a given phenotype compared to all the microchimeric immunophenotyped cells in the muscle tissue was significantly less than the proportion of autologous cells with that phenotype as a percentage of all autologous immunophenotyped cells (Table 1). In JIIM muscle, except for CD45RA+ and CD83+ cells, the proportion of microchimeric cells for each lineage as a proportion of all microchimeric cells was significantly less than the proportion of autologous cells of that same phenotype. The proportion of microchimeric cells that were CD45RA+ compared to all microchimeric cells was similar to, but not greater than, the proportion of autologous CD45RA+ cells compared to all autologous immunophenotyped cells. In MD biopsies, the proportion of microchimeric CD3+ and Class II+ cells as a percentage of all microchimeric cells was significantly less than their autologous counterparts, with a trend toward a lower frequency of microchimeric cells in CD8+ and CD123+ lineages compared to their autologous equivalents. In the control muscle tissue, significantly fewer microchimeric cells were found in CD3+, Class II+ and the CD25+ lineages compared to these autologous lineages as a proportion of the total autologous immunophenotyped cells, with a trend toward fewer microchimeric cells of the CD8+ and CD123+ lineages.

**Table 1:** Relationship between the frequencies of microchimeric versus autologous cells of a given immunophenotype

Phenotype	JIIM	MD	NIC
% Autologous CD3+ cells of total autologous^a^	6.5 ± 2.3	9.9 ± 2.5	4.8 ± 2.7
% Microchimeric CD3+ cells of total microchimeric cells^b^	0.3 ± 0.3	2.8 ± 1.8	10 ± 10
	*p* = 0.06 (T)	*p* = 0.04	*p* = 0.05
% Autologous CD4 cells of total autologous cells	16.8 ± 6.9	12.2 ± 5.3	8.4 ± 6.6
% Microchimeric CD4 cells of total microchimeric cells	2.2 ± 1.4	7.8 ± 2.0	0 ± 0
	*p* = 0.03	*p* = 0.23	*p* = 0.12
% Autologous CD8 cells of total autologous cells	20.4 ± 5.0	12.6 ± 3.2	5.1 ± 3.2
% Microchimeric CD8 cells of total microchimeric cells	1.1 ± 0.6	6.5 ± 2.3	0 ± 0
	*p* = 0.05	*p* = 0.09	*p* = 0.07
% Autologous Class II cells of total autologous cells	21.5 ± 8.1	51.6 ± 5.9	38.0 ± 11.0
% Microchimeric Class II cells of total microchimeric cells	1.7 ± 1.1	0.8 ± 0.7	20.0 ± 13.3
	*p* = 0.04	*p* < 0.001	*p* = 0.02
% Autologous CD20 cells of total autologous cells	8.5 ± 4.4	2.7 ± 2.5	0 ± 0
% Microchimeric CD20 cells of total microchimeric cells	0 ± 0	0 ± 0	0 ± 0
	*p* = 0.08	*p* = 0.3	*p* = NA
% Autologous CD83 cells of total autologous cells	0.1 ± 0.1	0.1 ± 0.1	0.2 ± 0.2
% Microchimeric CD83cells of total microchimeric cells	0 ± 0	0 ± 0	0 ± 0
	*p* = 0.18	*p* = 0.17	*p* = 0.17
% Autologous CD25 cells of total autologous cells	16.2 ± 5.1	9.5 ± 3.4	6.4 ± 2.8
% Microchimeric CD25 cells of total microchimeric cells	0 ± 0	7.7 ± 2.9	0 ± 0
	*p* = 0.01	*p* = 0.33	*p* = 0.02
% Autologous CD45RA cells of total autologous cells	2.3 ± 0.9	1.5 ± 0.4	3.2 ± 2.3
% Microchimeric CD45RA cells of total microchimeric cells	1.2 ± 1.1	0.9 ± 0.9	0 ± 0
	*p* = 0.24	*p* = 0.26	*p* = 0.10
% Autologous CD45RO cells of total autologous cells	6.3 ± 2.4	4.1 ± 1.1	9.0 ± 4.4
% Microchimeric CD45RO cells of total microchimeric cells	0 ± 0	0 ± 0	0 ± 0
	*p* = 0.03	*p* = 0.003	*p* = 0.06 (T)
% Autologous CD123 cells of total autologous cells	3.6 ± 1.1	1.9 ± 0.7	26.2 ± 12.2
% Microchimeric CD123 cells of total microchimeric cells	0.04 ± 0.02	0 ± 0	4.4 ± 2.3
	*p* = 0.04	*p* = 0.06 (T)	*p* = 0.06 (T)

Data are presented as mean ± standard error of the mean for each group

*JIIM* juvenile idiopathic inflammatory myopathy, *MD* muscular dystrophy, *NIC* noninflammatory controls; *NA* not available, *T* trend for significance

^a^Total autologous cells consists of all phenotyped cells with XY probes

^b^Total microchimeric cells consists of all cells with XX probes

### Do microchimeric cells mediate tissue repair?

Others have proposed that microchimeric cells might mediate tissue repair. In muscle diseases, they could contribute to regenerating myofibers. Thus, we hypothesized that in JIIM and MD the proportion of microchimeric myofiber nuclei would be enriched compared to autologous myofiber nuclei. We stained 15 sections for actin but found that the stain interfered with FISH, possibly due to the overabundance of actin within these muscle sections. Consequently, we subsequently quantified maternally derived myofiber nuclei based on the morphology of the nucleus and its location within the fiber (Fig. 2, *bottom*). The density of microchimeric myofiber nuclei was 0.01 ± 0.01 microchimeric cells/mm^2^ of muscle tissue in JIIM, 0.07 ± 0.03/mm^2^ in MD, and 0.02 ± 0.01/mm^2^ in controls. MD biopsies had significantly more microchimeric myofiber nuclei than JIIM (*p* = 0.018). The number of microchimeric myofiber nuclei did not differ between JIIM and controls or between MD and controls (*p* = 0.33 and *p* = 0.18, respectively). Like the phenotyped microchimeric cells, we found significantly fewer microchimeric muscle nuclei than autologous muscle nuclei in JIIM, MD and control biopsies. The proportion of microchimeric myofiber nuclei was significantly less than the proportion of autologous myofiber nuclei. The percentage of microchimeric myofibers compared with autologous myofibers from the same group was 0.6 ± 0.6 % in JIIM (*p* = 0.002), 3.6 ± 1.2 % in MD (*p* < 0.001) and 2.5 ± 1.4 % in controls (*p* = 0.0014) (see Fig. 2 for representative microchimeric myofibers).

## Discussion

Previously we found higher frequency and number of microchimeric cells in the peripheral blood and muscle tissue of JIIM patients compared to noninflammatory or healthy control subjects, suggesting that microchimeric cells may play a role in the pathogenesis of JIIM [4, 20]. One limitation in those studies was that we did not phenotype the microchimeric cells in the tissues; however, we did find microchimeric cells in the CD4+ and CD8+ lineages in peripheral blood [4]. The current study further investigated microchimeric cells in JIIM, compared with inflammatory but nonautoimmune muscle disease (MD) and with noninflammatory control muscle and examined the immunophenotypes of microchimeric cells in muscle tissue. Our goals were to determine whether microchimeric cells play a pathogenic role in disease and/or whether they are involved in tissue repair. Persistent microchimeric cells have been found in patients with other inflammatory diseases, suggesting that these cells might be recruited nonspecifically to sites of inflammation [7–10, 13, 14].

Compared with our prior study, we found a similar frequency of JIIM muscle tissue samples containing microchimeric cells and a similar overall quantity of microchimeric cells in JIIM muscle tissue [4]. Here, we also saw a similar concentration of microchimeric cells in MD tissues, suggesting that this finding is not specific to autoimmune diseases but may be generally associated with overt inflammatory muscle disease. This suggests that the number of inflammatory microchimeric cells recruited to the site of inflammation does not depend on the total number of inflammatory cells present. MD is a genetic disease caused by mutations in dystrophin inducing muscle fiber damage, with resultant muscle necrosis and tissue inflammation [21], whereas the cause of JIIM is unknown but presumed to result primarily from an autoimmune-mediated destruction of myofibers and muscle capillaries [17]. In both diseases, tissue destruction is mediated by T and B cells, and in JDM by dendritic cells [22–24]. We were surprised to find microchimeric cells also in a high proportion of control muscle tissues, in contrast to our prior study [4]; however, in the prior study, only one muscle section was examined, whereas in the present study, ten sections from each patient’s muscle tissue were examined. Microchimeric cells were not observed in every tissue section from a given patient and thus overall were considered to be a rare event.

To characterize the phenotype of microchimeric cells in JIIM and MD, we stained muscle tissue for various immunophenotypes. Overall, we detected few microchimeric cells within inflammatory phenotypes, and we did not find microchimeric cells in B cell or dendritic cell lineages, which are thought to be important in JIIM pathogenesis. Of the T cell immunophenotypes, generally the concentration of microchimeric cells was greater in MD tissues than in noninflammatory controls, and typically T cells or activated T cells were not elevated in JIIM muscle tissue. Microchimeric cells of a given phenotype did not occur more frequently than their autologous counterparts in JIIM or MD muscle tissue, compared to all microchimeric or immunophenotyped autologous cells. In terms of location, the endomysial and perivascular regions of the muscle showed similar numbers of microchimeric cells among the three groups. However, MD and JPM tissues were more likely to have microchimeric cells in the perimysium compared with controls. It is this sole finding that weakly suggests that microchimeric cells might play a pathogenic or reparative role.

We also found microchimeric cells in noninflammatory tissues and in myofibers, not only in infiltrating inflammatory cells, suggesting that these cells are resident in tissues regardless of inflammation. Tissues with maternal microchimeric cells include autoimmune and nonautoimmune thyroid [25], pancreas in type I diabetes [26], neonatal lupus heart muscle [27], tonsils and adenoids [28], cutaneous inflammatory diseases [29] and inflammatory bowel disease [30]. Normal tissues with maternal microchimeric cells include heart [27], tonsils/adenoids [28], skin diseases [29] and inflammatory bowel disease [30]; albeit at lower levels than diseased tissue of the same type, suggesting that a threshold number of microchimeric cells might be necessary to drive disease. However, in some instances microchimeric cells were found at comparable numbers in healthy tissues [6].

One hypothesis proposed by other investigators is that microchimeric cells might mediate tissue repair via microchimeric stem cells. Initially we stained for muscle fibers, but this staining procedure interfered with subsequent FISH. Therefore, we counted the numbers of microchimeric nuclei that were apparently myofibers, based on their morphological position and shape within the muscle fiber, and compared them to the numbers of autologous myofiber nuclei. We found significantly more microchimeric muscle nuclei in MD than JIIM muscle, but there were no significant differences between JIIM and controls. However, each disease class had significantly fewer microchimeric myonuclei than autologous muscle nuclei. One caveat is that we do not know how many myofibers were regenerated from autologous progenitors during the disease process [27], and this study was not designed to determine that. Overall, these results suggest that microchimeric myofibers are no more frequent than microchimeric inflammatory cells in diseased tissues, and that noninflammatory tissue resident in microchimeric myofiber nuclei are present at higher levels than observed in JIIM. This result suggests that microchimeric cells are not enriched in muscle tissue in any of these disease conditions, and that they do not mediate tissue repair at an augmented rate compared to autologous myofibers.

We stained one phenotype per FISH analysis for microchimeric cells in the tissue, but we did not stain for all cell phenotypes in the tissues. Consequently, we may have missed a microchimeric cell phenotype(s) that was noninflammatory, including muscle stem cells. We did not analyze all inflammatory phenotypes, such as macrophages, that are frequent in the inflammatory infiltrates of JIIM and MD muscle [22, 23], and some microchimeric cells did not match any of the phenotypes analyzed. The number of inflammatory cells in the JIIM samples was comparable to previously published data [22], and this suggests that the immunophenotyping was not underrepresenting inflammatory cell phenotypes. Furthermore, although we did not observe many differences in the phenotypic numbers of microchimeric cells between disease groups, only one or a small number of pathogenic microchimeric cells might be sufficient to cause disease; however, we believe this is unlikely as microchimeric cells were also present in the muscle tissue of noninflammatory controls. In addition, we did not examine the functionality of the microchimeric cells phenotyped and although microchimeric cells were found in inflammatory cells of the controls, they may have been quiescent, whereas in the inflammatory muscle diseases they may be activated, indicated by different cytokine profiles [31]. We did, however, investigate T cell activation markers, such as Class II, CD25, and memory T cell subsets, and did not detect more microchimeric cells in JIIM muscle compared to noninflammatory controls, and only MD had significantly more CD25 microchimeric cells. Finally, our sample sizes were relatively small and the study may have been underpowered to detect differences between groups.

Murine studies suggest that maternal microchimeric cells are able to manipulate the fetal immune system and promote the development of various Regulatory T cells (Tregs) that are tolerant toward the noninherited maternal antigens [32]. Additional studies using inbred mice have also reported the trafficking of alloreactive T cells in offspring and that the maternal cells can influence the fetal response to targeting specific tissues [33]. These studies were elegantly performed in mice, and whether this same phenomenon occurs to the same extent during human gestation leading to increased risk for autoimmune diseases is yet to be proved. Currently, no studies have been performed to determine whether maternal microchimerism of cells that carry noninherited shared epitopes place the JIIM cohort at a greater risk for disease, like they apparently do for rheumatoid arthritis [34]. HLA-DRB1*0301 in linkage with HLA-DQA1*0501 confers the highest susceptibility for JIIM; however, in contrast to previous studies [31], we found that HLA-DQA*0501 was not significantly associated with microchimerism [35]. Maternal microchimeric cell transfer is a frequent occurrence during fetal development [36], and these cells most likely distribute throughout all the tissues and become embedded in the bone marrow with the ability to differentiate into inflammatory cells or stromal cells upon cell damage. Thus, the presence of maternal microchimeric cells in tissues is not surprising in light of the high number of normal samples that were positive in our study and in other studies [6, 37]. Our data support the findings of Ye et al., who also reported their controls being positive for maternal microchimerism [6]. The presence of microchimeric cells in the control tissues without inflammation suggests that they are present either from fetal development if stromal in nature, or are inflammatory cells trafficking through the tissue. In contrast with Ye et al., we found the presence of some microchimeric T cells, albeit at very low percentages in our JDM samples. However, the direct role of maternal microchimeric cells in human disease is still not clear and against the complex genetic background of humans may mean that the direct role of these cells in autoimmunity may never be conclusively demonstrated.

## Conclusions

Overall, our results indicate that microchimeric cells likely do not play important pathogenic roles in autoimmune or inflammatory muscle disease, nor are they seemingly involved in mediating repair of muscle tissue. Once differentiated, where there is inflammation, the autologous cells, along with their microchimeric counterparts, appear to be recruited to those sites in like manner. This study suggests that microchimeric cells are not part of the autoimmune pathogenesis in JIIM or the primary inflammatory process in MD.

## Abbreviations

CD: cluster designation
FISH: fluorescence in situ hybridization
JDM: juvenile dermatomyositis
JIIM: juvenile idiopathic inflammatory myopathies
JPM: juvenile polymyositis
MD: muscular dystrophy
NIC: noninflammatory control
